# A Case of Extensive Abscess of the Liver; Effusion into the Peritoneum; Death; Autopsy

**Published:** 1853-05

**Authors:** J. R. Wilcox

**Affiliations:** Evansville, Ind.


Art. II.—*A Case of Extensive Abscess of the Liver; Effusion into the Peritoneum; Death; Autopsy.* By J. R. Wilcox, M. D., of Evansville, Ind.

Mr. B. aged 45 years, of irreproachable character and habits, medium stature, arrived in this city, September 4, 1852, seeking medical advice, on account of an enlargement in the right side.

He presented the following symptoms: a marked icteric hue of the conjunctiva and skin, much emaciation, slight cough, without expectoration, hurried respiration, almost suffocating dyspnea when in the recumbent position, hectic fever, with nocturnal perspiration, very frequent eructations, with occasional vomiting, capricious appetite, torpid bowels, stools very light, feet edematous, pulse one hundred and ten, soft. Stethoscopic examination showed a healthy condition of the lungs and heart.

Local symptoms were, pain complained of in the right side and region of the stomach, with an enlargement or tumor in the right side, elevating the false ribs from two to three inches, apparently four or five inches in diameter, not tender, except upon hard pressnre, no fluctuation, and no redness or discoloration of the integuments.

The lower lobe of the liver could be felt distinctly by well defined edges, low down in the abdomen, and was tender upon pressure; considerable distension of the abdomen.

He gave the following history of his illness: After great exposure upon the river during the extreme cold weather of January, 1852, he was seized, whilst standing in an insurance office, in Cincinnati, with rigors, vomiting, and an exceedingly acute pain in the right side and region of the stomach. He landed, in a few hours, at Rising Sun, and the vomiting and pain being unabated, he was there promptly attended by two or three physicians. After a few days, the symptoms were apparently controlled, but from that time he had some pain, occasional vomiting, and shortness of breath.

On the fourth day after his arrival here, I was called in haste to see him, and found him rapidly sinking. His skin was cold and clammy; he was almost pulseless and soon expired.

*Autopsy*, eighteen hours after death, by Drs. Wilcox and Mitchell. Witnessed by Drs. Walker, Byford, and Ham.

Upon laying the body bare, the right hypochondrium was elevated probably two inches above a normal position, but the tumor was not to be found.

Upon laying the abdomen open, the stomach was found in the umbilical region, and pus, in quantities almost beyond belief, was found in all parts of the abdominal cavity.

The liver being at least three times its natural size, had not become adherent to the walls of the abdomen, although I had endeavored to induce an external outlet, by blistering and fomentations; nor had the abscess opened upon the anterior surface of this viscus, but on the posterior, in the lobulus quadratus.

No abnormal appearance was found in the remaining viscera, except some slight traces of melanosis in the lungs.

January 15, 1853.

